# *Aspergillus fumigatus* responds to natural killer (NK) cells with upregulation of stress related genes and inhibits the immunoregulatory function of NK cells

**DOI:** 10.18632/oncotarget.12616

**Published:** 2016-10-12

**Authors:** Andreas Schneider, Michael Blatzer, Wilfried Posch, Ralf Schubert, Cornelia Lass-Flörl, Stanislaw Schmidt, Thomas Lehrnbecher

**Affiliations:** ^1^ Division of Pediatric Hematology and Oncology, Hospital for Children and Adolescents, Johann Wolfgang Goethe-University, Frankfurt, Germany; ^2^ Division of Hygiene and Medical Microbiology, Medical University of Innsbruck, Innsbruck, Austria; ^3^ Division of Pediatric Pulmonology, Allergology and Cystic Fibrosis, Hospital for Children and Adolescents, Johann Wolfgang Goethe-University, Frankfurt, Germany

**Keywords:** Aspergillus fumigatus, human natural killer cell, gene expression, perforin, cytokine, Immunology and Microbiology Section, Immune response, Immunity

## Abstract

Natural Killer (NK) cells are active against *Aspergillus fumigatus,* which in turn is able to impair the host defense. Unfortunately, little is known on the mutual interaction of NK cells and *A. fumigatus*. We coincubated human NK cells with *A. fumigatus* hyphae and assessed the gene expression and protein concentration of selected molecules. We found that *A. fumigatus* up-regulates the gene expression of pro-inflammatory molecules in NK cells, but inhibited the release of these molecules resulting in intracellular accumulation and limited extracellular availability. *A. fumigatus* down-regulatedmRNA levels of perforin in NK cells, but increased its intra- and extracellular protein concentration. The gene expression of stress related molecules of *A. fumigatus* such as heat shock protein *hsp90* was up-regulated by human NK cells. Our data characterize for the first time the immunosuppressive effect of *A. fumigatus* on NK cells and may help to develop new therapeutic antifungal strategies.

## INTRODUCTION

Natural Killer (NK) cells are lymphocytes of the innate immune system which are able to kill their target by cytotoxic molecules such as perforin or granzyme B or by death receptor-mediated apoptosis. In addition, NK cells enhance the activity of other arms of the host immune response such as of professional phagocytes, dendritic cells, and T cells through chemokines and cytokines such as interferon (IFN)-γ and granulocyte-macrophage colony-stimulating factor (GM-CSF) [1]. Although the term of NK cells came from their natural ability to kill tumor cells, it has been demonstrated that NK cells also exhibit cytotoxicity against virus-infected cells and bacteria, and play an important role in the antifungal host response such as against *Aspergillus fumigatus*, which is a major cause for morbidity and mortality in patients receiving chemotherapy for cancer or undergoing hematopoietic stem cell transplantation [2]. For example, it has been demonstrated that in neutropenic mice with invasive aspergillosis, depletion of NK cells increased the fungal load, whereas the transfer of activated NK cells resulted in greater pathogen clearance from the lungs [3]. From the pathogen's point of view, *A. fumigatus* produces a variety of proteins, such as proteinases or mycotoxins, all of which are important in the defense mechanisms of the fungus and tissue invasion, as they detoxify antifungal molecules produced by the host or break down the host defense barriers [4]. As we have demonstrated in an earlier study that *A. fumigatus* decreases the protein concentration of NK cell-derived IFN-γ in the supernatant [5], we investigated the influence of *A. fumigatus* on the expression of selected genes which are known to play a role in the antifungal activity of NK cells. Given the complex nature of the host-pathogen interaction, we also assessed the response of *A. fumigatus* to host immunity.

## RESULTS

### *A. fumigatus* increases mRNA levels of proinflammatory cytokines, but causes intracellular accumulation of these molecules and limits extracellular availability

**Figure 1:**
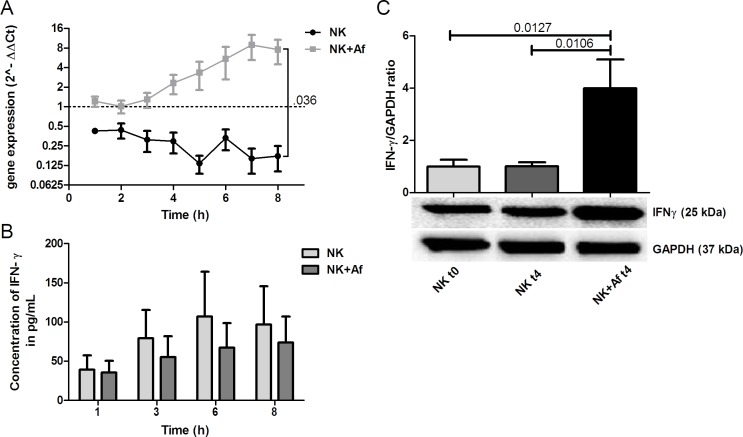
Gene expression and intra- and extracellular protein levels of IFN-γ **A.** Gene expression of interferon-gamma (IFN-γ; *IFNG*) in interleukin (IL)-2 pre-stimulated human NK cells co-incubated with *A. fumigatus* hyphae (grey squares, NK+Af) or incubated alone (black dots, NK). The X axis represents the time (hours); first assessment of transcript levels was performed at hour 1. The Y axis represents the relative fold-change of IFN-γ at specific time points to IFN-γ at time point 0 (dotted line; < 1 down-regulation, > 1 up-regulation). Squares and dots represent means, bars the standard error of means (*n* = 5). The *P* value represents the difference at time point 8 hours. **B.** Concentration of IFN-γ in the supernatant of NK cells incubated for up to 8 hours with *A. fumigatus* hyphae (dark-grey columns on the right, NK+Af)) or without the fungus (light-grey columns on the left, NK). The boxes represent means, the whiskers standard error (*n* = 5). **C.** Bottom: Western blot analysis of intracellular IFN-γ and GAPDH in NK cells incubated with *A. fumigatus* hyphae (NK + Af t4) or without the fungus (NK t4) for 4 hours and of the control at time point 0 (NK t0). Shown is one representative experiment out of a total of five independent experiments. Top: Calculated ratios of IFN-γ to GAPDH in NK cells at 0 hours (light-grey column on the left), NK cells incubated alone for 4 hours (dark-grey column in the center) and NK cells incubated with *A. fumigatus* hyphae for 4 hours (black column on the right). The boxes represent means, the whiskers standard error (*n* = 5).

When IL-2 prestimulated NK cells were incubated with *A. fumigatus* hyphae, mRNA levels of the pro-inflammatory molecule IFN-γ (*IFNG*) significantly increased over the time period of 8 hours (mean±SEM 7.6-fold±3.1-fold; *P* = .009) (Figure 1A). In contrast, mRNA levels of IFN-γ slightly decreased when NK cells were incubated alone (0.2-fold±0.1-fold; ns). As a result, at time point 8 hours, transcript levels of IFN-γ were 38-fold higher in NK cells coincubated with the fungus (*P* = .036) (Figure 1A and Table 1). However, despite the increased gene expression, the protein levels of IFN-γ measured after 8 hours in the supernatant of NK cells co-incubated with *A. fumigatus* were slightly lower than those of NK cells incubated alone (mean±SEM: 74±33 pg/mL *versus* 97±49 pg/mL) (Figure 1B and Table 1). To further investigate this phenomenon, we assessed the level of translation of IFN-γ. Western blot analysis revealed that the translation of IFN-γ occurred, but that the presence of *A. fumigatus* resulted in a significant intracellular accumulation of IFN-γ (mean±SEM of the IFN-γ/GAPDH ratio 4±1.1 in NK cells incubated with the fungus *versus* 1.0±0.15 in NK cells incubated alone; *P* = .0106) (Figure 1C). In contrast, when *A. fumigatus* in different concentrations was added to IFN-γ, the protein concentration was not affected, suggesting that fungal proteases do not play a major role in the low levels of IFN-γ in the supernatant (data not shown).

**Table 1:** Regulation of selected genes and extracellular protein levels of human Natural Killer cells in the presence or absence of *Aspergillus fumigatus*

human NK cells		gene expression			protein level	
	gene (molecule)	x-fold change	2^−ΔΔ Ct^; NK/NK+Af	*P*	pg/mL; NK/NK+Af	*P*
	Cytotoxic molecules					
	*PRF1* (perforin)	**-3**	2.1 ± 0.1/0.7 ± 0.1	< 0.0001	16229 ± 1814/17355 ± 1302	ns
	*GZMB* (granzyme B)	**-2.6**	0.8 ± 0.1/0.3 ± 0.1	0.001	2752 ± 544/4172 ± 314	ns
	Pro-inflammatory molecules					
	*IFNG* (interferon-gamma)	**+38**	0.2 ± 0.1/7.6 ± 3.1	0.036	97 ± 49/74 ± 33	ns
	*GMCSF* (GM-CSF)	**+4.6**	0.7 ± 0.1/3.2 ± 0.7	0.015	29 ± 14/28 ± 11	ns
	*MIP1A* (macrophage inflammatory protein 1α)	**+3.7**	0.6 ± 0.1/2.2 ± 0.5	0.016	1900 ± 768/1891 ± 784	ns
	*MIP1B* (macrophage inflammatory protein 1β)	**+3.7**	0.7 ± 0.1/2.6 ± 0.7	0.044	2402 ± 805/1923 ± 448	ns

Relative change of the mRNA expression of the gene of interest after 8 hours of (co-) incubation relative to the housekeeping gene and to time point at hour 0, respectively. The first value represents the results in NK cells incubated alone (NK), the second value the results in NK cells incubated with the fungus (NK+Af) (mean±SEM each). Negative values of “x-fold change” indicate down-regulation, positive values up-regulation.

Protein levels in the supernatant of the molecule of interest were assessed after 8 hours. The first value represents the results in NK cells incubated alone (NK), the second value the results in NK cells incubated with the fungus (NK+Af) (mean±SEM each). Shown are the results of at least five independent experiments.

**Figure 2:**
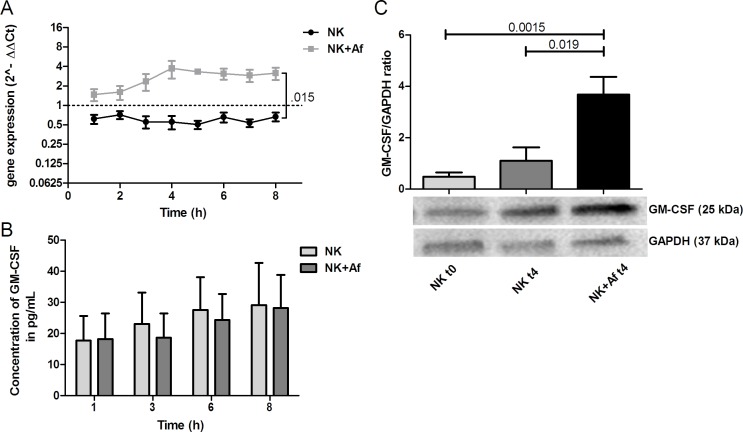
Gene expression and intra- and extracellular protein levels of GM-CSF **A.** Gene expression of granulocyte-macrophage colony stimulating factor (GM-CSF; *GMCSF*) in interleukin (IL)-2 pre-stimulated human NK cells co-incubated with *A. fumigatus* hyphae (grey squares, NK+Af) or incubated alone (black dots, NK). The X axis represents the time (hours); first assessment of transcript levels was performed at hour 1. The Y axis represents the relative fold-change of GM-CSF at specific time points to GM-CSF at time point 0 (dotted line; < 1 down-regulation, > 1 up-regulation). Squares and dots represent means, bars the standard error of means (*n* = 5). The *P* value represents the difference at time point 8 hours.**B.** Concentration of GM-CSF in the supernatant of NK cells incubated for up to 8 hours with *A. fumigatus* hyphae (dark-grey columns on the right, NK+Af)) or without the fungus (light-grey columns on the left, NK). The boxes represent means, the whiskers standard error (*n* = 5). **C.** Bottom: Western blot analysis of intracellular GM-CSF and GAPDH in NK cells incubated with *A. fumigatus* hyphae (NK + Af t4) or without the fungus (NK t4) for 4 hours and of the control at time point 0 (NK t0). Shown is one representative experiment out of a total of five independent experiments. Top: Calculated ratios of GM-CSF to GAPDH in NK cells at 0 hours (light-grey column on the left), NK cells incubated alone for 4 hours (dark-grey column in the center) and NK cells incubated with *A. fumigatus* hyphae for 4 hours (black column on the right). The boxes represent means, the whiskers standard error (*n* = 5).

Similar results were seen for GM-CSF. Compared to NK cells coincubated alone, the presence of *A. fumigatus* resulted in 4.6-fold increased transcript levels (*P* = .015), and increased intracellular protein concentrations (mean±SEM of the GM-CSF/GAPDH ratio 3.7±0.7 in NK cells incubated with the fungus *versus* 1.1±0.5 in NK cells incubated alone; *P* = .019), whereas extracellular protein concentrations did not change (29±14 pg/mL *versus* 28±11 pg/mL) (Figure 2 and Table 1).

Similarly, the gene expression of the pro-inflammatory molecules MIP-1α (*MIP1A*) and MIP-1β (*MIP1B*) significantly increased in the presence of *A. fumigatus* (3.7-fold each, *P* = .016 and .044, respectively) (Table 1). However, the up-regulation in the gene expression did not result in an increase of protein concentration in the supernatant (mean±SEM: 1900±768 pg/mL *versus* 1891±784 pg/mL and 2402±805 pg/mL *versus* 1923±448 pg/mL, respectively; Table 1).

### *A. fumigatus* down-regulates mRNA levels of perforin in human NK cells, but increases intracellular perforin concentrations and triggers the release of perforin

**Figure 3:**
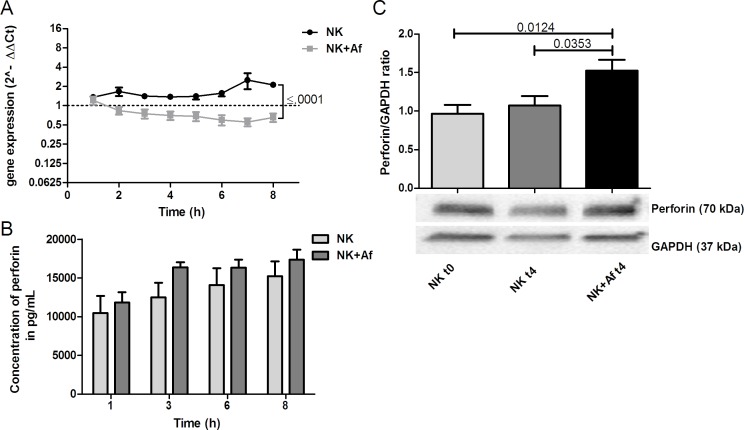
Gene expression and intra- and extracellular protein levels of perforin **A.** Gene expression of perforin (*PRF1*) in interleukin (IL)-2 pre-stimulated human NK cells co-incubated with *A. fumigatus* hyphae (grey squares, NK+Af) or incubated alone (black dots, NK). The X axis represents the time (hours); first assessment of transcript levels was performed at hour 1. The Y axis represents the relative fold-change of perforin at specific time points to perforin at time point 0 (dotted line; < 1 down-regulation, > 1 up-regulation). Squares and dots represent means, bars the standard error of means (*n* = 5). The *P* value represents the difference at time point 8 hours.**B.** Concentration of perforin in the supernatant of NK cells incubated for up to 8 hours with *A. fumigatus* hyphae (dark-grey columns on the right, NK+Af)) or without the fungus (light-grey columns on the left, NK). The boxes represent means, the whiskers standard error (*n* = 5). **C.** Bottom: Western blot analysis of intracellular perforin and GAPDH in NK cells incubated with *A. fumigatus* hyphae (NK + Af t4) or without the fungus (NK t4) for 4 hours and of the control at time point 0 (NK t0). Shown is one representative experiment out of a total of five independent experiments. Top: Calculated ratios of perforin to GAPDH in NK cells at 0 hours (light-grey column on the left), NK cells incubated alone for 4 hours (dark-grey column in the center) and NK cells incubated with *A. fumigatus* hyphae for 4 hours (black column on the right). The boxes represent means, the whiskers standard error (*n* = 5).

The presence of *A. fumigatus* significantly decreased the gene expression of the cytotoxic molecules perforin (*PRF1*) and granzyme B (*GZMB*) during the 8 hours of coincubation (*PRF1 P* = .001; *GZMB P* = .018). In contrast, mRNA levels of both cytotoxic molecules increased over time when NK cells were incubated alone (*P* = .016 for perforin, not significant for granzyme B). As a result, mRNA levels were significantly lower in NK cells after 8 hours of coincubation with *A. fumigatus* compared to NK cells incubated alone (*PRF1 P* < .0001; *GZMB P* = .001) (Figure 3A and Table 1).

Despite the decrease of the gene expression, *A. fumigatus* increased the protein concentration of perforin in NK cells (Figure 3C). Western blot analysis demonstrated a ratio of perforin to GAPDH of 1.5±0.14 (mean±SEM) after 4 hours of coincubation with the fungus *versus* 0.96±0.12 when NK cells were incubated alone (*P* = .0353). The high perforin levels in the supernatant of IL-2 pre-stimulated NK cells (mean±SEM 16,229±1814 pg/mL at time point 8 hours) were only slightly increased in the presence of *A. fumigatus* (mean±SEM 17,335±1302 pg/mL) (Figure 3B and Table 1).

### *A. fumigatus* up-regulates the gene expression of stress related molecules in the presence of human NK cells

**Figure 4:**
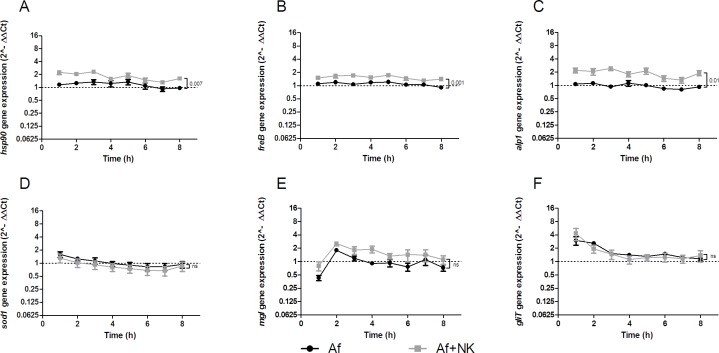
Gene expression profile of heat shock protein90 (*hsp90*) **A**, ferric chelate reductase (*freB*) **B.**, alkaline protease 1 (*alp1*) **C.**, superoxide dismutase (*sod1*) **D.**, mitogillin (*mgl*) **E.**, and gliotoxin (*gliT*) **F.** assessed in *A. fumigatus* co-incubated with pre-stimulated human NK cells (grey squares, Af+NK), or incubated alone (black dots, Af). The X axis represents the time (hours); the first assessment of transcript levels was performed at hour 1. The Y axis represents the relative fold-change (< 1 down-regulation, > 1 up-regulation) of the gene of interest at specific time points relative to time point 0 (dotted line; < 1 down-regulation, > 1 up-regulation). Squares and dots represent means, bars the standard error of means (*n* = 5). The *P* value represents the difference at time point 8 hours.

The transcript levels of the heat shock protein 90 (*hsp90*) were significantly higher when *A. fumigatus* was coincubated with NK cells (*P* = .007 at time point 8 hours) (Figure 4A and Table 2). Similarly, transcript levels of the fungal ferric chelate reductase (*freB*) were significantly increased upon exposure with NK cells at all time points investigated (*P* = .001 at time point 8 hours) (Figure 4B and Table 2).

**Table 2:** Regulation of selected genes in *Aspergillus fumigatus* in the presence or absence of human NK cells

*A. fumigatus*		gene expression		
	gene (molecule)	x-fold change	2^−ΔΔ Ct^; Af/Af+NK	*P*
	Heat shock response			
	*hsp70* (heat shock protein 70)	**+1.1**	0.9 ± 0.1/1.0 ± 0.1	ns
	*hsp90* (heat shock protein 90)	**+1.6**	1.0 ± 0.1/1.6 ± 0.1	0.007
	High affinity iron assimilation			
	*freB* (ferric chelate reductase)	**+1.6**	0.9 ± 0.0/1.4 ± 0.1	0.001
	Proteases/Peptidases			
	*alp1* (alkaline protease 1)	**+2.1**	0.9 ± 0.0/1.9 ± 0.3	0.01
	*dppIV* (dipeptidyl peptidase IV)	**+1.5**	0.6 ± 0.1/0.9 ± 0.1	ns
	*dppV* (dipeptidyl peptidase V)	**±1.0**	1.3 ± 0.2/1.3 ± 0.2	ns
	ROS^a^ detoxification			
	*sod1* (superoxide dismutase 1)	**±1.0**	0.9 ± 0.2/0.9 ± 0.2	ns
	*sod2* (superoxide dismutase 2)	**+1.4**	0.9 ± 0.2/1.3 ± 0.3	ns
	*sod3* (superoxide dismutase 3)	**+1.1**	1.5 ± 0.0/1.6 ± 0.2	ns
	*cat1* (catalase 1)	**±1.0**	0.6 ± 0.1/0.6 ± 0.1	ns
	*cat2* (catalase 2)	**+1.3**	1.3 ± 0.0/1.7 ± 0.4	ns
	*cycA* (cytochrome C)	**±1.0**	0.8 ± 0.1/0.8 ± 0.2	ns
	Mycotoxins			
	*mgl* (mitogillin)	**+1.6**	0.7 ± 0.1/1.1 ± 0.2	ns
	*gliT* (gliotoxin)	**+1.2**	0.7 ± 0.1/1.1 ± 0.2	ns

a ROS = reactive oxygen species

Relative change of the mRNA expression of the gene of interest after 8 hours of (co-) incubation relative to the housekeeping gene and to time point at hour 0, respectively. The first value represents the results in *A. fumigatus* incubated alone (Af), the second value the results in *A. fumigatus* incubated with NK cells (Af+NK) (mean±SEM each). Negative values of “x-fold change” indicate down-regulation, positive values up-regulation. Shown are the results of at least five independent experiments.

### Human NK cells differentially regulate the gene expression of fungal proteases, but do not have a major impact on fungal superoxide dismutases, catalases and mycotoxins

In the presence of human NK cells, *A. fumigatus* expressed significantly higher mRNA levels of alkaline phosphatase 1 (*alp1*) compared to those when the fungus was incubated alone (*P* = .01 after 8 hours of coincubation) (Figure 4C and Table 2). Transcript levels of the dipeptidyl peptidases IV and V, the superoxide dismutases 1-3, the catalases 1 and 2, and the mycotoxins gliotoxin and mitogillin were not significantly affected by the presence of NK cells (Figure 4D-4F and Table 2).

## DISCUSSION

Our data show that NK cells and *A. fumigatus* interact and mutually influence the gene expression of a variety of molecules of both host response and fungal survival pathways. *A. fumigatus* responds to NK cells with an up-regulation of stress related genes, whereas NK cells up-regulate in the presence of *A. fumigatus* the gene expression of pro-inflammatory cytokines such as IFN-γ or GM-CSF. This is not surprising, as these molecules play an important role in the antifungal host response, which is in line with results previously reported in NK cells coincubated with *C. albicans* [9]. IFN-γ stimulates the migration of phagocytes, enhances their phagocytic and oxidative killing activity of *A. fumigatus*, and is a signature cytokine of protective T_H_1 response [10]. Similarly, GM-CSF stimulates the production of professional phagocytes and enhances their antifungal activity [11]. We observed that the presence of *A. fumigatus* increases the gene expression of these molecules, which are also translated into proteins, but, at the same time, leads to an intracellular accumulation, which decreases the extracellular availability of these immunomodulators. Although we cannot exclude that there is an increased release of the proteins after our observation time period of 8 hours, it is important to note that NK represent the dominant source of IFN-γ during the early phase of infection when the adoptive immunity is not yet active as well as in individuals with a compromised T cell function [3, 12]. This immunosuppressive effect of *A. fumigatus* on NK cells has not been reported before, and might have deleterious consequences for the host. For example, it has been demonstrated in neutropenic mice with invasive aspergillosis that the depletion of NK cells resulted in reduced lung IFN-γ levels and increased lung fungal load that was independent of T and B cell lymphocytes [3]. However, when NK cells in IFN-γ deficient mice were depleted, no further increase in severity of the infection was seen. Finally, the transfer of activated NK cells from wild-type, but not from IFN-γ deficient mice led to greater pathogen clearance from the lungs of both IFN-γ deficient and wild-type recipients, all of which underlines the importance of NK cell derived IFN-γ in the host defense against *A. fumigatus*. The detailed analysis of this newly described effect of *A. fumigatus*, which is immunosuppressive by the intracellular accumulation of proinflammatory cytokines, is the current focus of our research and might help to develop better immunotherapeutic strategies in the future. Importantly, *A. fumigatus* and *R. oryzae* differently affect the concentration of IFN-γ and GM-CSF in the supernatant [5, 13], which suggests that each fungus exhibits specific immunosuppressive effects on NK cells and might offer new diagnostic tools. However, the interpretation of studies investigating the effect of fungi on NK cells has to consider whether and how the NK cells were prestimulated. Whereas we did not observe a significant difference in the gene expression profiles between unstimulated and IL-2 prestimulated NK cells (data not shown), prestimulation with other cytokines such as IL-12 or IL-18 may have different effects on NK cells [14]. Although our approach of prestimulating with IL-2 over 7-10 days is currently the standard setting for many clinical trials using NK cells, the effect of fungi on NK cells prestimulated with different protocols has to be assessed in the future [15, 16].

The transcript levels of perforin and granzyme B, which play a central role in NK cell mediated killing of fungi [5, 9, 13], increase in IL-2 prestimulated NK cells in the absence *of A. fumigatus*, but decrease in the presence of the fungus. The decrease of the gene expression of perforin with a concomitant increase of the intracellular protein concentration seems surprising, but can be explained by the fact that upon activation with *A. fumigatus*, NK cells rapidly upregulate the translation of preexisting mRNA of perforin [17]. The presence of *A. fumigatus* also leads to a slight increase of the perforin levels in the supernatant due to degranulation, an effect which has also been observed in NK cells coincubated with *R. oryzae* or *C. albicans* [13, 18]. However, as IL-2 prestimulation of NK cells alone already results in relatively high extracellular perforin levels and enhanced cytotoxicity, *A. fumigatus* only had a marginal effect on the extracellular perforin concentration.

The presence of NK cells resulted in an upregulation of the expression of several stress-related fungal genes, such as the heat shock protein *hsp90*. This is plausible, as stress response pathways are essential for adaptation to hostile environments which *Aspergillus* hyphae encounter when coincubated with human NK cells. Hsp90 of *A. fumigatus* is at the center of a complex network involving calcineurin, lysine deacetylases and other client proteins, which orchestrate compensatory repair mechanisms of the cell wall in response to the stress induced by antifungals [19]. Interestingly, disrupting Hsp90 circuitry by different means such as Hsp90 inhibitors potentiates the activity of the antifungal compound caspofungin [19], suggesting that further studies should investigate the influence of this approach in order to increase the efficacy of antifungal strategies. We also observed a significant increase in the gene expression of the ferric reductase *freB*, which has recently been identified as an important enzyme in filamentous fungi for the adaptation to iron starvation [20]. Our results are in line with a recent study in *C. albicans* which reports that the perforin-induced reduction of iron availability results in the upregulation of the gene expression of Csa 2, which is involved in the uptake of iron of human hemoglobin [9]. Iron plays a key role in fungal growth and survival, and animal studies have shown a benefit of iron chelators as a therapeutic strategy in invasive aspergillosis [21].

Proteins secreted by the fungus such as peptidases and proteases play an important role in the pathogenesis of invasive fungal disease as they help to invade host tissue and to manifest invasive growth. In this respect, we observed an up-regulation of the transcript levels of the fungal alkaline protease 1 (asp f13, *alp1*) when the pathogen was coincubated with prestimulated human NK cells. This molecule is involved in the pathogenesis of asthma and other lung diseases associated with epithelial barrier impairment, as it infiltrates the bronchial submucosa and disrupts airway smooth muscle cell-extracellular matrix interactions [22]. In addition, Alp1 plays a role in T_H_1/T_H_2 differentiation, helps *A. fumigatus* to evade from the host complement attack by its complement-degrading activity and might therefore be an interesting target in the development of antifungal therapy [23, 24].

Interestingly, *A. fumigatus* seems to be able to specifically adapt the response to the host immune cell. For example, co-incubation of the fungus with airway epithelial cells leads to an upregulation of genes counteracting oxidative stress [25], whereas we did not observe an alteration of the gene expression of the catalases *cat1* and *cat2* in the presence of NK cells, in which reactive oxygen species do not play a major role in the clearance of pathogens.

As a number of molecules involved in the antifungal activity of NK cells have previously been described, we decided to analyze the gene expression and protein concentrations of these molecules in more detail. However, future studies analyzing the transcriptome and proteome need to complement our results characterizing the complex host-pathogen interaction.

In conclusion, we describe for the first time that *A. fumigatus* exhibits an immunosuppressive effect on human NK cells by inhibiting the release of pro-inflammatory cytokines, whereas *A. fumigatus* up-regulates in the presence of NK cells stress-related genes. The results of our study might help to develop approaches to strengthen specific antifungal host immune responses, which ultimately could improve outcome of invasive aspergillosis.

## MATERIALS AND METHODS

### Isolation and cultivation of primary human NK cells

Primary human NK cells were isolated from peripheral blood from different healthy volunteers. Cells were isolated by negativ selection using the EasySep^®^ Human NK Cell Enrichment Kit (StemCell Technologies, Grenoble, France) according to the manufacturer's instructions. Viability and purity of the isolated CD56^+^CD3^−^ NK cells were ≥92% and ≥98%, respectively, as determined by flow cytometry (Canto II, Beckton Dickinson, San Jose, USA). Isolated NK cells were cultivated for 10 days in RPMI (Gibco, Paisley, UK) supplemented with 5% human frozen plasma (German Red Cross Blood Donor Service Baden-Wuerttemberg-Hessen, Frankfurt, Germany) and stimulated with recombinant human interleukin (rhIL)-2 (1000 U/mL; Novartis, Basel, Switzerland) every three days. The protocol was approved by the local ethics committee.

### Preparation of *A. fumigatus*

The *A. fumigatus* strain AF4215 (MYA 1163; American Type Culture Collection) was grown on Sabouraud glucose agar (BD Bioscience, San Jose, USA) at 37°C for 2-3 days. Conidia were harvested by gently washing the surface with PBS (Gibco) supplemented with 0.05% Tween-20 (Sigma-Aldrich, Taufkirchen, Germany). The number of the conidia was estimated in a Neubauer slide (LO-Laboroptik, Friedrichsdorf, Germany). Resting conidia were immediately used or stored at 4°C. For preparation of *A. fumigatus* hyphae, 1.25×10^4^ resting conidia were plated in 48-well flat-bottom cell culture plates (Nunc, Langenselbold, Germany) and incubated in 500 μL Yeast Nitrogen Base (Sigma-Aldrich) supplemented with (D)-Glucose (Sigma-Aldrich) at 37°C for 17 hours to allow formation of mycelium.

### Co-incubation of *A. fumigatus* and human NK cells and preparation of total RNA

A total of 2.5×10^5^ NK cells were co-incubated with *A. fumigatus* mycelium in 500 μL NK cell culture medium in 48-well flat-bottom culture plates (Nunc) for up to 8 hours at 37°C._._

For the preparation of RNA, samples were collected immediately prior to co-incubation and thereafter every hour during co-incubation for up to 8 hours. Total RNA of both NK cells and the fungus was immediately extracted by means of the RNeasy Plus Micro Kit (Qiagen, Hilden, Germany) according to the manufacturer's instructions. For extraction of fungal RNA, the cell wall of *A. fumigatus* was cracked by three freeze-thaw cylces, consisting of heating to 95°C and direct freezing in liquid nitrogen to −196°C. The isolated RNA was dissolved in RNase-free water and the concentration and purity of the samples were determined using a NanoDrop^®^ ND-1000 spectrophotometer (Thermo Scientific, Wilmington, USA). For the reverse transcription of the mRNA of *A. fumigatus* and NK cells to cDNA, the High Capacity RNA-to-cDNA Kit (Applied Biosystem, Foster City, USA) was used according to the manufacturer's instruction.

### Primers and amplicons for human NK cells

Representative target-specific NK cell gene sequences were obtained by the NCBI GeneBank. Both primer pairs and UPL TaqMan^®^ probes were selected from conserved regions of respective specific sequences based on the intron-spanning method of the online Universal Probe Library Assay Design Center (Roche, Mannheim, Germany). Primers were purchased from MWG (Eurofins Genomics, Ebersberg, Germany), UPL probes from Roche (Supplementary Table 1). Amplicon lenghts ranged between 66 and 123 bp with an annealing temperature of 60°C. Uniqueness was tested with the NCBI BLAST search.

### Primers and amplicons for *A. fumigatus*

Sequences for *A. fumigatus* were retrieved from the Central Aspergillus Data REpository site [6]. Gene identities and primer sequences are listed in Supplementary Table 2. Primers were designed to span exon-intron boarders, and amplicon lengths ranged between 104 and 138 bp with an annealing temperature of 58°C. Primer efficiency was analyzed by ten-fold serial dilutions for human and *Aspergillus* specific primer-setups.

### Quantitative real-time PCR for human NK cells

Real-time PCR for human NK cell genes was performed in a total volume of 20 μL and for each gene analyzed in technical duplicates. The mastermix contained 1x ABsolute qPCR ROX Mix (Thermo Scientific), primer pairs and probes 250 nM each, and 1 μL of template cDNA. The qRT-PCR was performed in a iQ5 platform (Bio-Rad, Munich, Germany) using the following thermal cycling conditions: 95°C for 12 min for initial denaturation, followed by 55 cycles of 95°C for 15 sec, and 60°C for 60 sec. All PCR assays included a housekeeping gene of human NK cells as reference [glyceraldehyde 3-phosphate dehydrogenase (*GAPDH*)] [7]. The Ct-values were generated by the Bio-Rad iQ5 Software with a threshold of 100. The 2^−ΔΔCt^-method was used to calculate the regulation of the genes [8].

### Quantitative real-time PCR for *A. fumigatus*

qRT-PCR was performed from cDNA using SsoFast EvaGreen Supermix (Bio-Rad) in a Bio-Rad CFX96 real time PCR detection system. The total reaction volume was 20 μL, containing forward and reverse primers 400 nM each and 2 μl of cDNA template. Reactions were performed in technical duplicates. The parameters consisted of an initial denaturation at 98°C for 30 sec, and 40 cycles of 98°C for 5 sec, 58°C for 5 sec followed by a melt curve analysis to confirm the specificity of the PCR products. PCR assays included a housekeeping gene of *A. fumigatus* cells as reference [beta-tubulin; (*TUBB*)]. Data were analysed using the 2^−ΔΔCt^-method [8].

### Assessment of protein concentrations in the supernatant

Concentrations of IFN-γ were assessed by a commercially available enzyme-linked immunoabsorbent assay (ELISA; BioLegend, San Diego, USA), of all other molecules by cytokine bead array (CBA; BD Biosciences) according to the manufacturers' instructions. The detection limit was 5.6 pg/mL for IFN-γ, 40 pg/mL for granzyme B, and 10 pg/mL for all other molecules.

### Western blot analysis

Prestimulated human NK cells were coincubated with or without hyphae of *A. fumigatus* for 4 hours. NK cells at time point 0 hours served as control. Cells were lysed with RIPA Lysis and Extraction Buffer Kit (Thermo Scientific) containing HALT Phosphatase and Protease Inhibitor Cocktails (both Thermo Scientific). Proteins were separated by SDS-PAGE using 4-15% Mini Protean TGX Precast Protein Gels (Bio-Rad). Proteins were blotted onto a nitrocellulose membrane which was blocked with 5% skim milk-PBS solution. Mouse monoclonal antibodies against human IFN-γ (1:200; LifeSpan BioSciences, Seattle, USA), human perforin (1:1000, LifeSpan BioScience), human GM-CSF (1:1000, R&D Systems), and human GAPDH (1:20000; Biolegend) were used as primary antibodies, goat anti-mouse IgG-HRP antibody (1: 100000; Abcam, Cambridge, UK) as secondary antibody. For visualization, Gel Doc™ XR+ System (Bio-Rad) and Amersham ECL Select Western Blotting Detection Reagent (GE Healthcare Life Sciences, Little Chalfont, UK). For comparison of intracellular protein levels, the ratio of the protein of interest and GAPDH was calculated by determination of relative band intensities using Image Lab 5.0 software (Bio-Rad).

### Statistical analysis

Data were analyzed using GraphPad Prism (version 5.04 for Windows, GraphPad Software, La Jolla California, USA). The unpaired *t*-test was used to compare different groups. A *P*-value (two-tailed) of < .05 was considered to be statistically significant.

## SUPPLEMENTARY MATERIALS TABLES


