# Supplementary material for: Aspergillus fumigatus responds to natural killer (NK) cells with upregulation of stress related genes and inhibits the immunoregulatory function of NK cells
Source: Oncotarget. 2016 Oct 12;7(44):71062–71. doi: 10.18632/oncotarget.12616 (PMC5342063; doi:10.18632/oncotarget.12616)
Supplement: Supplementary file 1 [file oncotarget-07-71062-s001.pdf]

Aspergillus fumigatus responds to natural killer (NK) cells with upregulation of stress related genes and inhibits the immunoregulatory function of NK cells

Supplementary Material

Supplementary Table 1: Human NK cell genes and primers for qRT-PCR

gene	gene Bank no.	ID	primer sequence (5´-3´)	amplicon size (bp)
glyceraldehyde 3-phosphate dehydrogenase (*GAPDH*)	NM_002046.3	GAPDH_for<br>GAPDH_rev	AGC CAC ATC GCT CAG ACA C<br>GCC CAA TAC GAC CAA ATC C	66
perforin (*PRF1*)	NM_005041.4	Perf_for<br>Perf_rev	CAT CTG TGT AGC CGC TTC TCT<br>GCA GCA GCA GGA GAA GGA T	81
granzyme B (*GZMB*)	NM_004131.4	GZMB_for<br>GZMB_rev	GAG ACG ACT TCG TGC TGA CA<br>GAG ACG ACT TCG TGC TGA CA	122
interferon-gamma (*IFNG*)	NM_000619	IFNg_for<br>IFNg_rev	GGC ATT TTG AAG AAT TGG AAA G<br>TTT GGA TGC TCT GGT CAT CTT	112
granulocyte-macrophage colony stimulating factor (*GMCSF*)	NM_000758.3	GMCSF_for<br>GMCSF_rev	CAA ACA TTT CTG AGA TGA CTT CTA CTG<br>GTC CTC GGA TGC TAA CCT CTA C	109
macroghage inflammatory protein 1α (*MIP1A*)	NM_002983.2	CCL3_for<br>CCL3_rev	GGC TCT CTG CAA CCA GTT CT<br>AAT CTG CCG GGA GGT GTA G	82
macroghage inflammatory protein 1β (*MIP1B*)	NM_002984.2	CCL4_for<br>CCL4_rev	CTC TCC AGC GCT CTC AGC<br>ACC ACA AAG TTG CGA GGA AG	93

Supplementary Table 2: *Aspergillus fumigatus* genes and primers for qRT-PCR

gene	gene locus	ID	primer sequence (5´-3´)	amplicon size (bp)
beta tubulin (*TUBB*)	AFUA_7G00250	tub1-f<br>tub1-r	CTG TCG GTC CAC CAG TTG<br>GGT TGA GAT CGC CGT ACG	122
heat shock protein 70 (*hsp70*)	AFUA_1G07440	hsp70-f<br>hsp70-r	AGG TCA AGG CTA CCG CTG<br>AGA GCA CGT GCG TTG GTG	127
heat shock protein 90 (*hsp90*)	AFUA_6G04830	hsp90-f<br>hsp90-r	TAC TGG TGG GTC ATT CTC TG<br>CTT GCG TTG GCC AGC TTG	134
ferric chelate reductase (*freB*)	AFUA_1G17270	freB-f<br>freB-r	AGC ATC GTG CTC TGC CTG<br>AAG AGA GGA TCC CGG TGC	112
alkaline protease 1 (*alp1*)	AFUA_4G11800	alp1-f<br>alp1-r	TTC TGT CGT CGC TGC TGG<br>AGT TGG AGA AGG AGG CGC	122
dipeptidyl peptidase IV (*dppIV*)	AFUA_4G09320	dppIV-f<br>dppIV-r	GTG CTC TGG GCT ACC AAC<br>GAG CGT ACT GGA TGT CGC	130
dipeptidyl peptidase V (*dppV*)	AFUA_2G09030	dppV-f<br>dppV-r	GGA CGC GAA GGT CTC AAC<br>AAC TGG AGG ATC CGC TCG	123
superoxide dismutase 1 (*sod1*)	AFUA_5G09240	sod1-f<br>sod1-r	CGT GGC TTC CAT GTC CAC<br>CCA AGG TCA CCG ACA TGG	128
superoxide dismutase 2 (*sod2*)	AFUA_4G11580	sod2-f<br>sod2-r	GCT TGG CTT GTC AGG GAC<br>TAA GCA TGC TCC CAG GCG	118
superoxide dismutase 3 (*sod3*)	AFUA_1G14550	sod3-f<br>sod3-r	ACC AGG ATC CGG TGA CCG<br>GTT CCA GAT GCC CTT GGC	113
catalase 1 (*cat1*)	AFUA_3G02270	cat1-f<br>cat1-r	CTG GCC CGT GAT GT TCA C<br>ACG GCG TGG ATC AGA TCG	131
catalase 2 (*cat2*)	AFUA_6G03890	cat2-f<br>cat2-r	CGA ATG ACC GCC GAA CTG<br>TTC CGA ATG CGC CAG TGC	126
cytochrome C (*cycA*)	AFUA_2G13110	cycA-f<br>cycA-r	AGG GAT ACG CCT ACA CCG<br>GCC TTC TTC AGA CCA CCG	133
mitogillin (*mgl*)	AFUA_5G02330	mgl-f<br>mgl-r	CCA CAG CCG TGT CTG TTC<br>CTG TAT AGA AGC CGC TTG TC	121
gliotoxin (*gliT*)	AFUA_6G09740	gliT-f<br>gliT-r	GAT GTC GCC AAG ACC GTC<br>TCA AGG CGA GCT GCT TGG	118
